# Methylation and Acetylation Enhanced the Antidiabetic Activity of Some Selected Flavonoids: In Vitro, Molecular Modelling and Structure Activity Relationship-Based Study

**DOI:** 10.3390/biom8040149

**Published:** 2018-11-15

**Authors:** Qamar Uddin Ahmed, Murni Nazira Sarian, Siti Zaiton Mat So’ad, Jalifah Latip, Solachuddin Jauhari Arief Ichwan, Nurlaili Najmie Hussein, Muhammad Taher, Alhassan Muhammad Alhassan, Hanisuhana Hamidon, Sharida Fakurazi

**Affiliations:** 1 Department of Pharmaceutical Chemistry, Faculty of Pharmacy, International Islamic University Malaysia, Kuantan 25200, Pahang DM, Malaysia; dszaiton@iium.edu.my (S.Z.M.S.); lailinajmie@yahoo.com (N.N.H.); alhasanma@yahoo.com (A.M.A.); 2 Laboratory of Vaccines and Immunotherapeutics, Institute of Bioscience, Universiti Putra Malaysia, Serdang 43400, Selangor, Malaysia; sharida@upm.edu.my; 3 School of Chemical Sciences and Food Technology, Faculty of Science and Technology, Universiti Kebangsaan Malaysia, Bangi 43600, Selangor, Malaysia; 4 Faculty of Dentistry, International Islamic University Malaysia, Kuantan 25200, Pahang DM, Malaysia; solachuddin@iium.edu.my; 5 Department of Pharmaceutical Technology, Faculty of Pharmacy, International Islamic University Malaysia, Kuantan 25200, Pahang DM, Malaysia; mtaher@iium.edu.my (M.T.); hanisuhana.hamidon@gmail.com (H.H.); 6 Department of Human Anatomy, Faculty of Medicine and Health Sciences, Universiti Putra Malaysia, Serdang 43400, Selangor, Malaysia

**Keywords:** flavonoids, type 2 diabetes mellitus, RIN-5F pancreatic cells, 3T3-L1 pre-adipocytes, adipokines, SAR, molecular docking

## Abstract

Flavonoids have been reported to exert antihyperglycemic effects and have potential to enhance the current therapy options against type 2 diabetes mellitus. However, the structure activity relationships (SAR) studies of flavonoids against this disease have not been thoroughly comprehended. Hence, in the present study, 14 structurally related flavonoids viz. wogonin, techtochrysin, norwogonin, isoscutellarein, hypolaetin, kaempferol, quercetin, methyl ether of wogonin, acetate of wogonin, acetate of norwogonin, 8-hydroxy-7-methoxyflavone, chrysin, (+)-catechin and (-)-epicatechin were taken into account for in vitro antidiabetic evaluation. Cell viability of RIN-5F pancreatic cells and 3T3-L1 pre-adipocyte cells was initially tested, then an insulin secretion assay of RIN-5F as well as adipogenesis and glucose uptake measurements of adipocyte were investigated. Subsequently, protein expressions study through adipokines measurement (leptin, adiponectin, TNF-α, RBP-4) via enzyme-linked immunosorbent assay (ELISA) kit, Western blotting analysis against GLUT4 and C/EBP-α as well as molecular docking against GLUT1 were analyzed. The results from cell culture antidiabetic assays (insulin secretion, adipogenesis, and glucose uptake), protein expressions and molecular docking pointed that the methoxy group at position C-8 is responsible for antidiabetic property of selected flavonoids via glucose uptake mechanism indicated by up regulation of GLUT4 and C/EBP-α expressions. The mechanism could be enhanced by the addition of an acetate group at C-5 and C-7 of the flavone skeleton.

## 1. Introduction

Type 2 diabetes mellitus (T2DM) is a common disease responsible for significant morbidity and mortality around the world. According to the American Diabetes Association, this disease is one of the major metabolic disorders that continue to present a significant health problem worldwide and mostly associated with chronic disturbances in protein, carbohydrate and lipid metabolisms. It is a serious debilitating disease that has now reached an epidemic proportion and is on the rise at an alarming rate [1].

**Figure 1:**
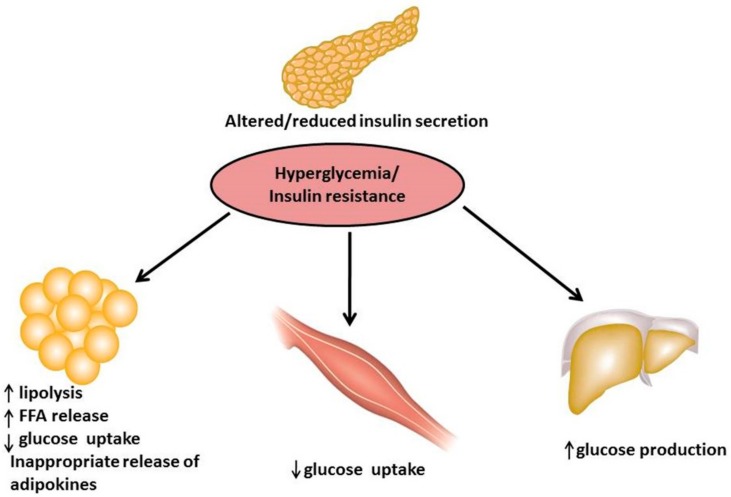
Pathophysiology of type 2 diabetes mellitus (T2DM).

Figure 1 depicts the pathophysiology of T2DM where pancreas dysfunction causes altered/reduced insulin secretion, increased lipolysis and free fatty acid (FFA) levels, low glucose uptake and inappropriate adipokines (i.e., retinol binding protein (RBP)-4, leptin, tumor necrosis factor (TNF)-α,) released by adipose tissues. Under these conditions, the glucose-sensitive tissues (i.e., skeletal muscle and adipocytes) are unable to accommodate the increase in glucose concentration. In addition, a low level of insulin stimulates the liver to increase glucose production into the blood stream [2]. On the other hand, hyperglycaemia develops in type 2 diabetes and occurs when there is an imbalance of glucose production (e.g., aberrant hepatic glucose production during fasting) and glucose intake (e.g., food consumption) as opposed to insulin-stimulated glucose uptake in target tissues, mainly skeletal muscles, fat tissue and liver [3]. This condition contributes directly to both insulin resistance and impairs pancreatic β-cell function, which is described as glucose toxicity. Persistent glucose release prolongs the hyperglycaemic environment, leading ultimately to T2DM [4].

Flavonoids are a class of plant secondary metabolites that have well categorized structure function relationships. They are abundantly found in vegetables, fruits, herbs, tea, cocoa, soy, red wine and other plant food and beverage products. Flavonoids are well reputed as antioxidants in nature, however, they also can act as an antihyperglycemic agent as has been demonstrated by several researchers [5,6,7,8,9,10]. It has also been reported that flavonoids can function as insulin secretagogue or insulin mimetic [11], probably by influencing the pleiotropic mechanisms, to attenuate the diabetic complications [12]. Furthermore, flavonoids have been found to stimulate glucose uptake in peripheral tissues and regulate the activity and/or expression of the rate-limiting enzymes that are involved in carbohydrate metabolism pathway [9,13]. Numerous researchers have investigated the potent activity of flavonoids against diabetes mellitus [14,15]. As a result, flavonoids are currently considered as one of the most promising and significantly important class of natural products to enrich the current therapy choices against type-2 diabetes mellitus.

Although there are only a few studies on the structure activity relationship (SAR) analysis of flavonoids isolated from different medicinal plants as far as their antidiabetic potential is concerned [8,9,14,16], the SAR studies to understand the comprehensive mechanism of antidiabetic potential of flavonoids are yet to be extensively carried out. Hence, this study was aimed at investigating the role of methoxy and acetate groups in flavonoids and its analogs owing to the fact that the antidiabetic activity of flavonoids is affected by the presence of different functional groups. Therefore, attempts were made to investigate their structure relationship and correlation for in vitro and in silico antidiabetic effects. It is envisioned that further advancement of this research work may lead to the development of nutritional products and semi-synthetic analogs that retain substantial antidiabetic capacity with minimal adverse effects.

## 2. Materials and Methods

### 2.1. Chemicals, Reagents and Cells

8-hydroxy-7-methoxyflavone, (+)-catechin, (-)-epicatechin, quercetin (control), ascorbic acid, trolox, ABTS+ radical, potassium persulphate, xanthine, xanthine oxidase, anhydrous potassium carbonate, anhydrous sodium sulphate, acetic anhydride, pyridine, 2,2-diphenyl-1-picrylhydrazyl (DPPH) were purchased from Sigma Aldrich (Singapore). 2,4,6-Tris (2-pyridyl)-s-triazine (TPTZ, 99%), iron (III) chloride hexahydrate and sodium acetate were purchased from Sigma-Aldrich (St. Louis, MO, USA). Rin-5F pancreatic cell line and 3T3-L1 preadipocyte cells were bought from ATCC, Manassas, VA, USA. 96 well plates, 24 well plates and RPMI-1640 media were bought from Gibco, (San Diego, CA, USA). ELISA kits (mouse RBP-4, mouse leptin, mouse TNF-α, mouse adiponectin) were bought from Cusabio Biotech Co., Ltd., Houston, TX, USA. An insulin secretion enzyme-linked immunosorbent assay (ELISA) kit was purchased from Elabscience, Wuhan, China. For Western blotting, 10% SDS TGX stain free™ FastCast acrylamide gel kits, Immun-Blot^®^ polyvinylidene difluoride (PVDF), non-fat dried milk were purchased from Bio-Rad, Santa Rosa, CA, USA. Primary antibodies (β-actin, GLUT-4, PPAR-α, C-ebp/α) were bought from ElabScience, Wuhan, China. Secondary antibody HRP-conjugated goat anti-rabbit immunoglobulin (Ig) was purchased from GeneTex, Irvine, CA, USA. Chemiluminescence, Chemi-Lumi One L and Tween-20 were bought from Nacalai-tesque, Kyoto, Japan.

### 2.2. Compounds’ Isolation and Derivatisation

**Figure 2:**
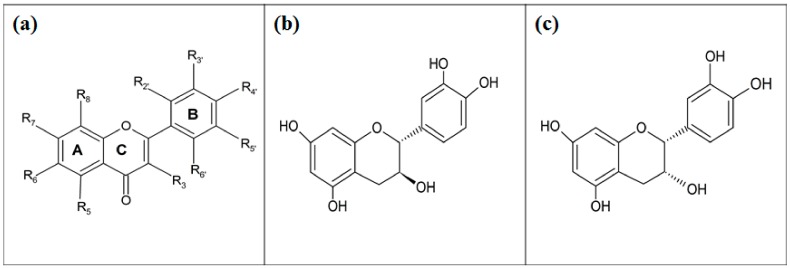
Structures of the tested flavonoids; (**a**) MN1-MN12, (**b**) (+)-Catechin (MN13), (**c**) (-)-Epicatechin (MN14). Wogonin (MN1) → R_5_ = OH, R_7_ = OH, R_8_ = OMe. Methyl ether of wogonin (MN2) → R_5_ = OMe, R_7_ = OMe, R_8_ = OMe. Acetate of wogonin (MN3) → R_5_ = OAc, R_7_ = OAc, R_8_ = OMe. Techtochrysin (MN4) → R_5_ = OH, R_7_ = OMe. 8-Hydroxy-7-methoxyflavone (MN5) → R_7_ = OMe, R_8_ = OH. Chrysin (MN6) → R_5_ = OH, R_7_ = OH. Norwogonin (MN7) → R_5_ = OH, R_7_ =OH, R_8_ = OH. Acetate of norwogonin (MN8) → R_5_ = OAc, R_7_ = OAc, R_8_ = OAc. Isoscutellarein (MN9) → R_5_ = OH, R_7_ = OH, R_8_ = OH, R_4′_ = OH. Hypolaetin (MN10) → R_5_ = OH, R_7_ = OH, R_8_ = OH, R_3′_ = OH, R_4′_ = OH. Kaempferol (MN11) → R_3_ = OH, R_5_ = OH, R_7_ = OH, R_4′_ = OH. Quercetin (MN12) → R_3_ = OH, R_5_ = OH, R_7_ = OH, R_3′_ = OH, R_4′_ = OH.

Three flavones namely; wogonin, norwogonin and techtochrysin were isolated from leaves of *Tetracera indica* using the method described by Alhassan et al. [17]. Similarly, two flavones (hypolaetin and isoscutellarein) and two flavonols (kaempferol and quercetin) were isolated from leaves of *Tetracera scandens* following the procedure described by Sarian et al. [9]. Wogonin and norwogonin were subjected to semi-synthesis to afford their methyl ether and acetate derivatives [9]. These compounds’ structures were characterized by spectroscopic analysis (nuclear magnetic resonance (NMR), infrared (IR), ultraviolet (UV), mass spectrometry). Their spectral data were further compared with the previously reported spectral data of the similar compounds already isolated from different plants to ensure their correct structures (Figure 2).

### 2.3. In Vitro Antidiabetic Evaluation

#### 2.3.1. Cell Viability Assay (RIN-5F Pancreatic Cells and 3T3-L1 Adipocyte Cells)

The RIN-5F pancreatic cells originated from islet cell tumor of rat (ATCC^®^ CRL-2058™) were seeded at density of 1.0 × 10^4^ cells/well in 96-wells plate and allowed for attachment at 37 °C for 72 h [18]. The cells were exposed to the compounds (0.78–100 μg/mL) for 24 h at 37 °C in humidified 5% CO_2_ atmosphere. The 3T3-L1 adipocyte cells originating from mouse embryos (ATCC^®^ CL-173™) were seeded in 96-wells plate at density of 2.0 × 10^5^ cells/well, and allowed for attachment overnight at 37 °C. The medium was removed and was replaced with 100 μL medium (pre-treated with compounds at different concentrations from 0.78–100 μg/mL) for 48 h at 37 °C in humidified 5% CO_2_ atmosphere. At the termination of the culture, both cells were rinsed with 1X phosphate buffered saline (PBS) and 20 μL of MTT solution (5 mg/mL) was added to each well, and the cells were then cultured for 4 h. Then, 100 μL of DMSO was added to each well to dissolve formazan crystals and further incubated for 1 h. The optical density (OD) was measured at 570 nm, with reference at 630 nm by a microplate reader (Infinite M200 Nanoquant Tecan, Männedorf, Switzerland). Cells viability was estimated according to the stated equation given below [19]:

$$\;\mathrm{Viability}\;\left(\%\right)=\left[\frac{\left(\mathrm{Absorbance}\;\mathrm{sample}-\mathrm{Absorbance}\;\mathrm{blank}\right)}{\left(\mathrm{Absorbance}\;\mathrm{control}-\mathrm{Absorbance}\;\mathrm{blank}\right)}\right]\times 100\;$$

#### 2.3.2. Insulin Secretion Assay

RIN-5F cells were used to evaluate insulin secretory activity [18]. A quantity of 2.0 × 10^5^ cells/well was seeded in 24-well plates in RPMI 1640 medium. After incubation for 72 h, the medium was removed. The cells were pre-incubated with 0.5 mL Krebs Ringer bicarbonate (KRB) buffer supplemented with 1.1 mM glucose, for 40 min at 37 °C. The buffer was then replaced with 0.5 mL KRB buffer solution treated with tested flavonoids, glibenclamide (200 μM) as positive control and non-treated cells as the negative control. After incubation for 3 h at 37 °C, aliquots in all wells were withdrawn and centrifuged at 1000 rpm for 5 min. The concentration of insulin in the media was determined by ELISA kits (Elabscience, Wuhan, China) in accordance with the manufacturer’s instructions and optical density was measured immediately at 450 nm by a microplate reader (Infinite M200 Nanoquant Tecan, Männedorf, Switzerland).

#### 2.3.3. Adipogenesis Assay

For adipogenesis, the protocols described by Park et al. [20] and Zhu et al. [21] were followed with some modifications [22]. Briefly, 5.0 × 10^5^ 3T3-L1 preadipocytes were seeded in a 96-wells flat bottom microplate and induced the adipogenic cocktail after 2 days of the 100% of confluency of the cells. Then, the adipogenic cocktail (0.5 mM IBMX, 1 μM dexamethasone, 10 μg/mL insulin in DMEM) and tested compounds (25, 12.5 and 6.25 μg/mL) were added and incubated for 2 days. Untreated cells were served as control and 10 μM rosiglitazone was served as positive control. After that, the media was replaced to an insulin media which contained of 10 μg/mL insulin in Dulbecco’s modified Eagle medium (DMEM) only. Then, at day 4, the media was changed with DMEM in every 2 days until day 8. Once differentiation of cells had completed, the cells were fixed with 10% formalin for 1 h at room temperature. Then, the cells were washed 3 times with PBS and stained with 100 μL of freshly prepared Oil Red O (6 oil red:4 dH_2_O) per well for 1 h. Cells were again washed with dH_2_O. After 5 min, 100 μL isopropanol was added per well to lyse the lipid droplets. Finally, the absorbance was read by microplate reader (Infinite M200 Nanoquant Tecan, Männedorf, Switzerland) at 540 nm.

#### 2.3.4. Fluorescence Glucose Uptake Measurement Assay

The fluorescence glucose uptake (2-NBDG) measurement method described by Hasan et al. [22] was strictly followed with some modifications. Briefly, 5.0 × 10^5^ of 3T3-L1 preadipocytes were seeded using a 96-well black fluorescence plate and allowed to grow up to 100% of confluency. After 2 days, the cells were treated with adipogenic cocktail to differentiate into mature adipocytes. Upon completing the differentiation of 3T3-L1 preadipocyte (on day 8), the cells were made to starve by incubating in serum and glucose-free DMEM for two days. After that, the 3T3-L1 adipocytes were further incubated with 80 μM 2-NDBG and tested compounds with 10 μg/mL insulin for another two days. 10 μM rosiglitazone was served as positive control. The cultures carefully were washed with 1X PBS to eliminate the remaining 2-NBDG. Lastly, the encapsulated fluorescence in the cells was measured by fluorescence microplate reader (Perkin Elmer, Rodgou, Germany) at an excitation wavelength of 485 nm and emission wavelength of 535 nm. The 100% of specific 2-NBDG uptake was determined by following equation:

 Specific Absorbance=Absorbance of insulin induced 2NDBG−Absorbance of non insulin induced 2NDBG 

### 2.4. Molecular Antidiabetic Evaluation

#### Leptin, Adiponectin, Tumor Necrosis Factor-A (Tnf-A) and Retinol Binding Protein-4 (Rbp-4) Assays

After the completion of the adipogenesis process on day 8, the 3T3-L1 adipocytes were treated with tested flavonoids at 40 μg/mL for 48 h. Subsequently, supernatants were collected and further used to evaluate leptin, adiponectin, TNF-α, and RBP-4 adipocytokines (Cusabio Biotech Company, Limited). Adipocytokines levels were measured using ELISA kits at the wavelength of 450 nm in accordance with the manufacturer’s instructions. Each experiment was performed in triplicate, and the results were presented as the means ± SD. Groups of data were compared using one-way analysis of variance (ANOVA) and Tukey’s multi comparison test.

### 2.5. Western Blotting Assay

Protein content was quantified according to Nouroozi et al. [23] with some modifications with bovine serum albumin chosen as a standard. 3T3-L1 adipocyte treated with tested flavonoids, cell lysates were run on 10% SDS TGX stain free™ FastCast acrylamide kits. Separated proteins were transferred to Immun-Blot^®^ polyvinylidene difluoride (PVDF), 0.22 μm membranes (Bio-Rad, Santa Rosa, CA, USA) at 90 volts with cooling, in transfer buffer, Tran-Blot^®^ (Bio-Rad, Santa Rosa, CA, USA) for 2 h. At the conclusion of the transfer, the membranes were then blocked for 1 h with 5% non-fat dried milk in 20 mmol L^−1^ Tris–HCl, Tween20, 0.15 mol L^−1^ NaCl buffer (TBS-Tween), pH 8. The membranes were then incubated with primary antibodies 1:1000 (GLUT-4, C/EBP-α and β-actin goat anti-mouse as the loading reference) in 5% non-fat dried milk in TBS-Tween buffer for 24 h, then washed several times with TBS-tween buffer. The secondary antibody used for all membranes was horseradish peroxidase (HRP)-conjugated goat anti-rabbit IgG (GeneTex, Irvine, CA, USA) at a dilution of 1:1000, 5% non-fat dried milk in TBS-Tween buffer for 1 h, followed by several washes as above at a dilution of 1:500 [24]. The membranes were then developed with chemiluminescence, Chemi-Lumi One L (Nacalai-Tesque, Kyoto, Japan). Proteins were visualised using gel documentation (Bio-Rad, Santa Rosa, CA, USA) and further analyzed by using Image J software [25].

### 2.6. In-Silico Molecular Docking of Glucose Transporter 1 (Glut 1)

A molecular docking approach was applied in this study to investigate the potential molecular targets of tested flavonoids. Molecular docking software named AutoDock 4.2 was used to execute the docking process. Proteins namely GLUT 1 was used as target for all tested flavonoids. The crystal structures of the proteins were retrieved from the protein data bank (PDB) and were used as receptors to perform the molecular docking study. Software including Molecular Graphics Library MGLTools 1.5.6, AutoDock Tools 4.2 [26] and Discovery Studio Visualizer 4.0 [27] were used for the molecular docking experiments. The 3D structure for GLUT1 (PDB ID: 5EQI) was obtained from the protein data bank [28]. Crystallographic waters and co-crystallized ligand were removed, polar hydrogens were added to a macromolecule by using AutoDock 4.2, after which the structure was saved in PDBQT file format that contains a protein structure with hydrogen in all polar residues. The 2D structures of the compounds were built with ChemDraw and converted to 3D format using ChemBio3D and saved in PDB file format. The ligands were then prepared for docking by computing the charges and the structures were saved in PDBQT file format via AutoDock 4.2 [29].

A molecular docking experiment of ligands was carried out using crystal structure of human glucose transporter GLUT1 co-crystallized with cytochalasin B (PDB ID: 5EQI). The size and centre coordinates of the grid box were first validated by re-docking the co-crystallized ligand on binding sites of the receptor. The rotational bonds of the ligands were treated as flexible while those of the protein were kept rigid. Grid boxes were fixed around the cytochalasin B binding site using the ligand as the grid box centre. The box size was set to 60, 60 and 60 Å3 (x, y and z respectively) and the grid spacing to 0.375 Å. The grid maps for atoms were calculated for all the ligands. A genetic algorithm (GA) was used for searching and the population size was set to 150, 100 runs and 5 million energy evaluations. Potential ligands (based on fluorescent glucose uptake and Western blotting result) were then docked into the active site of the enzyme using the grid box parameter obtained from the redocking of co-crystallized ligand as reference.

### 2.7. Statistical Analysis

Data were collected and expressed as the mean ± standard deviation (SD) of three independent experiments and analysed for statistical significance from each control. The data were tested for statistical differences by one-way ANOVA followed by Tukey or Dunnet’s multiple comparison tests of MiniTab (version 18). The criterion for significance was set at *p* < 0.05.

## 3. Results

### 3.1. In Vitro Antidiabetic Evaluation

#### 3.1.1. Viability Study of RIN-5F Pancreatic Cells

**Figure 3:**
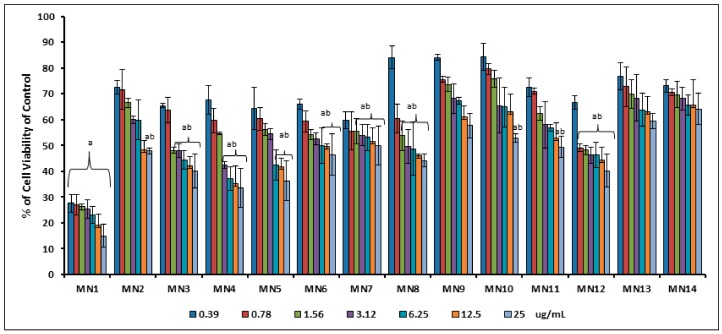
The percentage of cell viability of RIN-5F ranging from 0.39–25 μg/well in 1 h. Mean ± standard deviation (S.D.), *n* = 3, small letters represent Dunnet’s test. Mean values that do not share a letter are significantly different (*p* < 0.05).

Figure 3 displays the percentage of cell viability of RIN-5F pancreatic measured by MTT assay from 25.0–0.39 μg/well in 1 h of tested flavonoids treatment for screening purpose. Results showed that wogonin (MN1) revealed significant toxicity at all concentrations (*p* < 0.05). Meanwhile, acetate of wogonin (MN3), acetate of norwogonin (MN8) and quercetin (MN12) showed the IC_50_ value at 6.25 μg/well, whereas isoscutellarein (MN9), hypolaetin (MN10), (+)-catechin (MN13) and (-)-epicatechin (MN14) showed good cell viability at all concentrations. From this result, the lowest concentration (0.39 μg/well) was chosen for the insulin secretion assay for all the tested flavonoids.

#### 3.1.2. Insulin Secretion Assay (Rin-5F Pancreatic Cells)

**Figure 4:**
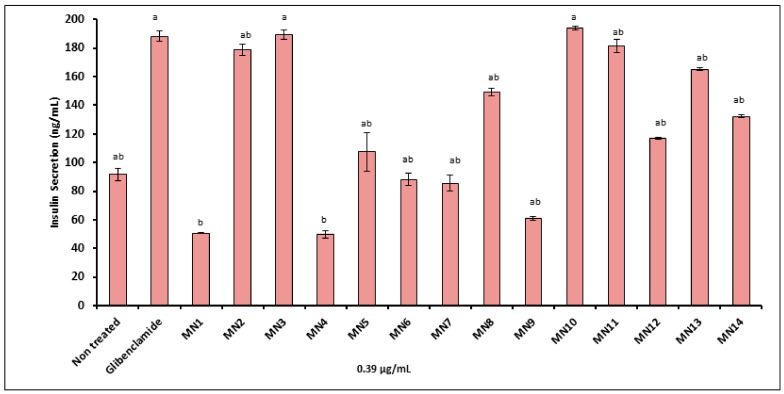
The effect of the flavonoid on insulin secretion activity of RIN-5F pancreatic cells. Mean ± S.D., *n* = 3, means that do not share a letter are significantly different (*p* < 0.05).

Figure 4 portrays the effect of tested flavonoids on the insulin secretion activity of RIN-5F pancreatic cell line as compared to glibenclamide (positive control). The graph shows that, at 0.39 μg/mL, acetate of norwogonin (MN3) and hypolaetin (MN10) showed the highest insulin release, similar to glibenclamide (positive control), followed by methyl ether of wogonin (MN2), kaempferol (MN11), (+)-catechin (MN13), acetate of norwogonin (MN8), (-)-epicatechin (MN14), quercetin (MN12), 8-hydroxy-7-methoxyflavone (MN5), chrysin (MN6), norwogonin (MN7) and isoscutellarein (MN9). Meanwhile, wogonin (MN1), and techtochrysin were shown to hamper the insulin secretion as compared to non-treated control (*p* < 0.05).

#### 3.1.3. Viability Study of 3T3-L1 Preadipocyte Cells

**Figure 5:**
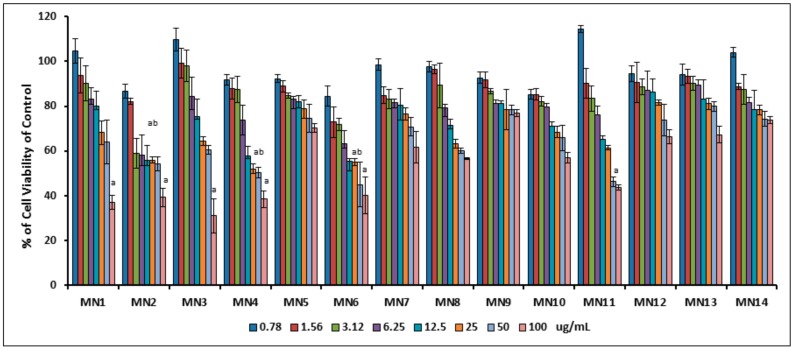
The percentage of cell viability of 3T3-L1 preadipocyte cells measured by MTT assay, ranging from 0.78 to 100 μg/well for 48 h. Small letters represent Dunnet’s test. Mean ± S.D., *n* = 3, means values that do not share a letter are significantly different (*p* < 0.05).

Figure 5 demonstrates the percentage of cell viability of 3T3-L1 preadipocyte cells measured by MTT assay from 0.78–100 μg/well for 48 h. Result exhibited that the cell viability was decreased as the concentration of the tested flavonoids increased. At 0.78 μg/well, cells showed 100% viability or more of all groups (MN1-MN14) as compared to the non-treated control. As the concentration slightly increased from 1.56 to 25.0 μg/well, the viability was decreased above the IC_50_ values. IC_50_ value of chrysin (MN6) and norwogonin (MN7) began to reach at 50 μg/well, and at 100 μg/well, wogonin (MN1), methyl ether of wogonin (MN2), acetate of wogonin (MN3), techtochrysin (MN4), 8-hydroxy-7-methoxyflavone (MN5) and kaempferol (MN11) showed toxicity effect while norwogonin (MN7), acetate of norwogonin (MN8), isoscutellarein (MN9), hypolaetin (MN10), quercetin (MN12), (-)-epicatechin (MN14) and (+)-catechin (MN13) showed IC_50_ values higher. From this result, concentrations of treatments below 25.0 μg/well were considered as safe to be used for further study.

#### 3.1.4. Adipogenesis (Oil Red O Staining)

**Figure 6:**
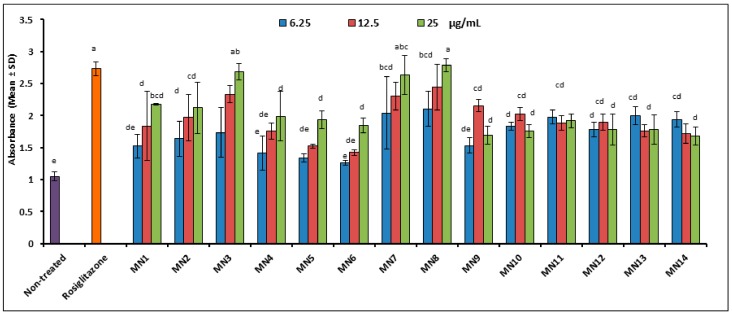
Quantification of lipid droplet after treatment. Small letters represent Tukey’s test. Mean ± S.D., *n* = 3, means values that do not share a letter are significantly different (*p* < 0.05).

Figure 6 shows the quantification of lipid droplet from adipogenesis after flavonoids treatment and were compared to rosiglitazone as the positive control. Results showed that at 25.0 μg/well, acetates of wogonin (MN3) and norwogonin (MN8) displayed the highest value of lipid droplet stained by oil red O, followed by norwogonin (MN7), wogonin (MN1), and methyl ether of wogonin (MN2). However, techtochrysin (MN4), 8-hydroxy-7-methoxyflavone (MN5), and chrysin (MN6) also showed good lipid droplet formations as compared to non-treated group, (*p* < 0.05). These groups exhibited increased in lipid droplet formation in dose dependent manner. It can be observed that isoscutellarein (MN9), hypolaetin (MN10), kaempferol (MN11), quercetin (MN12), (+)-catechin (MN13) and (-)-epicatechin (MN14) showed average lipid droplet formation, however not in a dose-dependent manner, as compared to other flavonoids groups.

**Figure 7:**
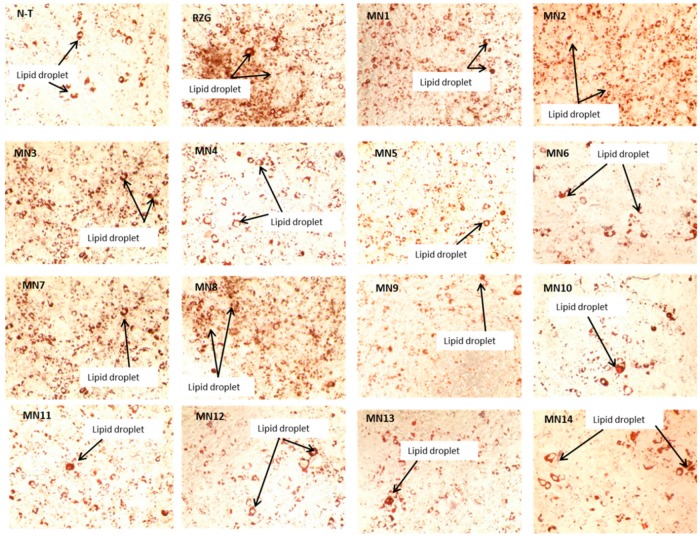
Lipid droplets formations of 3T3-L1 adipocyte cells stained with Oil red O. Magnification 40×. (NT: non-treated, MN1: wogonin, MN2: methyl ether of wogonin, MN3: acetate of wogonin, MN4: techtochrysin, MN5: 8-hydroxy-7-methoxyflavone, MN6: chrysin, MN7: norwogonin, MN8: acetate of norwogonin, MN9: isoscutellarein, MN10: hypolaetin, MN11: kaempferol, MN12: quercetin, MN13: (+)-catechin and MN14: (-)-epicatechin).

Figure 7 illustrates the microscopic observation under 40× magnification of lipid droplet stained by oil red O stain by all tested flavonoids at 25 μg/well. It can be observed that rosiglitazone at 10 μM (positive control) showed the most lipid droplet followed by acetate of norwogonin (MN8), acetate of wogonin (MN3), norwogonin (MN7), wogonin (MN1) and methyl ether of wogonin (MN2). The rest of the groups showed average lipid droplet formation as compared to the non-treated group (N-T).

#### 3.1.5. Fluorescent Glucose [2-NDBG] Uptake Measurement

**Figure 8:**
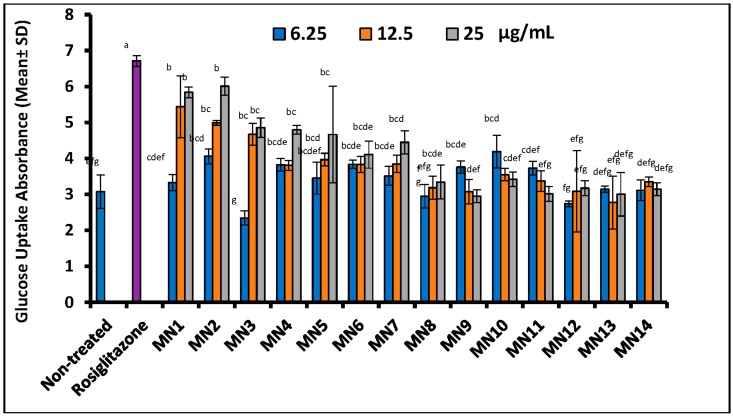
The effect of flavonoids on glucose uptake measurement of adipocyte cells. Small letters represent Tukey’s test. Mean ± S.D., *n* = 3, means values that do not share a letter are significantly different (*p* < 0.05).

Figure 8 illustrates the effect of flavonoids on glucose [2-NDBG] uptake measurement of 3T3-L1 adipocyte cells. The result showed that at 6.25 μg/mL, only hypolaetin (MN10) showed statistically significant glucose uptake as compared to non-treated control (*p* < 0.05). At 12.5 and 25 μg/mL, wogonin (MN1), methyl ether of wogonin (MN2), acetate of wogonin (MN3), techtochrysin (MN4), 8-hydroxy-7-methoxyflavone (MN5) and norwogonin (MN7) showed statistically significant glucose uptake measurement as compared to the non-treated group (*p* < 0.05). Rosiglitazone (positive control) displayed the highest absorbance of the fluorescence at concentration of 10 μM (*p* < 0.05). It is observable that, wogonin (MN1), methyl ether of wogonin (MN2), acetate of wogonin (MN3), techtochrysin (MN4), 8-hydroxy-7-methoxyflavone (MN5), chrysin (MN6), norwogonin (MN7) and acetate of norwogonin (MN8) showed the glucose [2-NDBG] uptake in a dose-dependent manner, whereas, isoscutellarein (MN9), hypolaetin (MN10), kaempferol (MN11), quercetin (MN12), (+)-catechin (MN13), and (-)-epicatechin (MN14) did not follow the same trend of glucose uptake. This consistent trend has further validated the lipid droplet formation (adipogenesis) result.

### 3.2. Molecular Antidiabetic Evaluation

#### 3.2.1. Measurement of Adipokines (Leptin, Adiponectin, Tumor Necrosis Factor-A (TNF-A), Retinol Binding Protein-4 (RBP-4)) of 3T3-L1 Adipocyte Cells Upon Flavonoids Treatment

**Figure 9:**
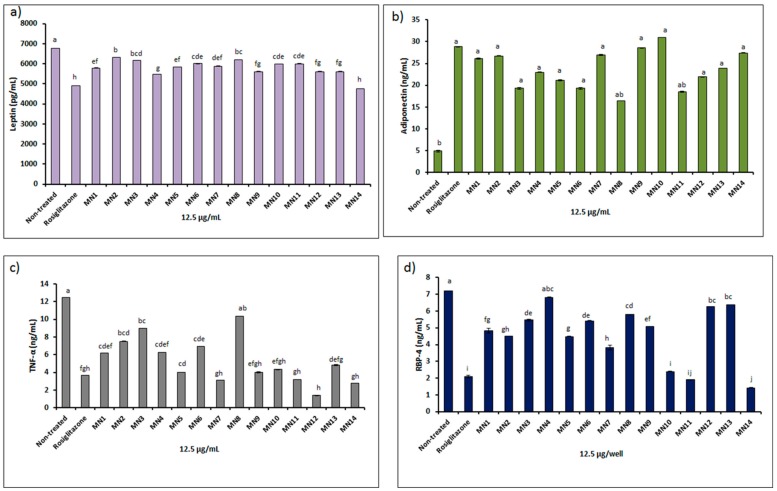
Adipokines measurement of adipocyte after flavonoids treatment at 12.5 μg/mL; (**a**) leptin, (**b**) adiponectin, (**c**) TNF-α, (**d**) RBP-IV. Mean ± S.D., *n* = 3, mean values that do not share a letter are significantly different (*p* < 0.05).

Figure 9 presents the adipokines measurement originated from 3T3-L1 adipocyte cells viz., leptin (a), adiponectin (b), TNF-α (c) and RBP-IV (d) of all tested flavonoids via enzyme-linked immunosorbent assay (ELISA). For leptin measurement, at 12.5 μg/mL, result of all groups of tested flavonoids showed statistically reduced in leptin concentration (pg/mL) as compared to the non-treated group (*p* < 0.05). However, (-)-epicatechin (MN14) showed the lowest reduction similar to positive control, rosiglitazone (10 μM), followed by techtochrysin (MN4), isoscutellarein (MN9), quercetin (M12), (+)-catechin (MN13), wogonin (MN1), 8-hydroxy-7-methoxyflavone (MN5), norwogonin (MN7), chrysin (MN6), hypolaetin (MN10), kaempferol (MN11), acetate of wogonin (MN3), acetate of norwogonin (MN8), and methyl ether of wogonin (MN2), respectively.

- For adiponectin measurement, at 12 μg/mL, except for acetate of norwogonin (MN8) and kaempferol (MN11), result of the tested flavonoids showed statistically increased in adiponectin concentration (ng/mL) as compared to the non-treated group (*p* < 0.05). Hypolaetin (MN10), showed the highest concentration, followed by isoscutellarein (MN9), norwogonin (MN7), (-)-epicatechin (MN14), methyl ether of wogonin (MN2), wogonin (MN1), (+)-catechin (MN13), techtochrysin (MN4), quercetin (MN12), 8-hydroxy-7-methoxyflavone (MN5), acetate of wogonin (MN3) and chrysin (MN6), respectively.
- For tumor necrosis factor, i.e., α (TNF-α), at 12 μg/mL, except for acetate of norwogonin (MN8), the result of the tested flavonoids displayed statistically decreased in TNF-α concentration (ng/mL) as compared to the non-treated group (*p* < 0.05). Quercetin (MN12) showed the lowest concentration of TNF-α, followed by (-)-epicatechin (MN14), norwogonin (MN7), kaempferol (MN11), (+)-catechin (MN13), wogonin (MN1), techtochrysin (MN4), chrysin (MN6), methyl ether of wogonin (MN2) and the least concentration was shown by the acetate of wogonin (MN3), respectively.
- Subsequently, for retinol binding protein IV (RBP-IV), at 12 μg/mL, except for techtochrysin (MN4), result of the tested flavonoids exhibited statistically reduced in RBP-IV concentration (ng/mL) as compared to the non-treated group (*p* < 0.05). (-)-epicatechin (MN14) showed the lowest concentration, followed by kaempferol (MN11), hypolaetin (MN10), norwogonin (MN7), 8-hydroxy-7-methoxyflavone (MN5), methyl ether of wogonin (MN2), wogonin (MN1), isoscutellarein (MN9), chrysin (MN6), acetate of norwogonin (MN3), acetate of norwogonin (MN8), quercetin (MN12), and the least concentration was displayed by (+)-catechin (MN13), respectively.

#### 3.2.2. Western Blotting

**Figure 10:**
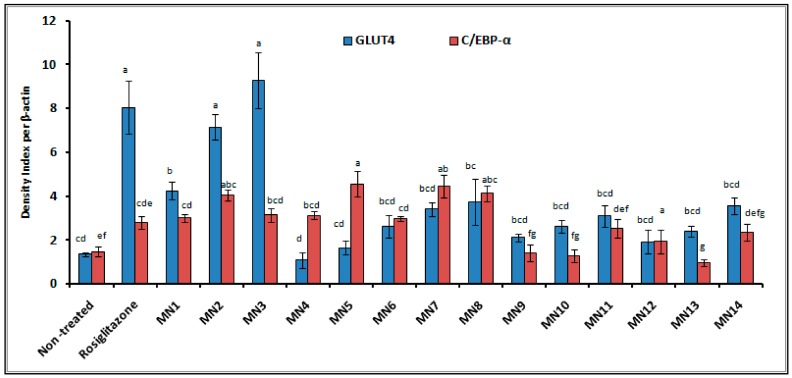
The density of protein bands of GLUT4 and C/EBP-α per β-actin. Mean ± S.D., *n* = 3, mean values that do not share a letter are significantly different (*p* < 0.05).

**Figure 11:**
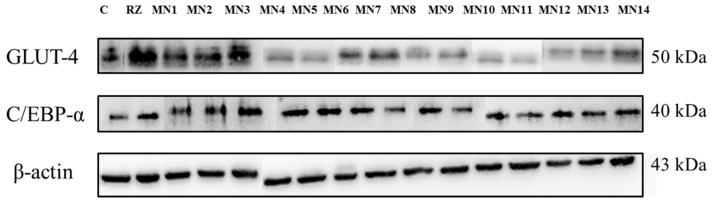
Image of protein bands of GLUT4, C/EBP-α and β-actin (internal control) on polyvinylidene difluoride (PVDF) membrane.

Figure 10 shows the density index of proteins band of glucose transporter 4 (GLUT4) and CCAAT/enhancer-binding protein α (C/EBP-α) projected from the image of protein bands that segregated based on the molecular weight on the polyvinylidene difluoride membrane (Figure 11). β-actin was used as the internal standard to normalize the band of protein interest. For GLUT4 protein, acetate of wogonin (MN3), methyl ether of wogonin (MN2) and wogonin (MN1) showed significant upregulation expression (*p* < 0.05). Techtochrysin (MN4) and 8-hydroxy-7-methoxyflavone (MN5) showed no significant difference with non-treated group, while the rest of the flavonoids showed slightly upregulation expression, however, these results were not significant as compared to non-treated group (*p* > 0.05). For the expression of C/EBP-α, 8-hydroxy-7-methoxyflavone (MN5) showed the highest expression, followed by norwogonin (MN7), acetate of norwogonin (MN8), methyl ether of wogonin (MN2), techtochrysin (MN4), wogonin (MN1), and chrysin (MN6) (*p* < 0.05). The rest of the flavonoids showed downregulation of C/EBP-α expression (*p* > 0.05).

### 3.3. In Silico Molecular Docking (GLUT 1)

Table 1 shows the docking scores against GLUT1 indicated by free binding energies. Lower binding energy depicts better ligand receptor interaction as well as higher docking score. Acetate of wogonin (MN3) showed the highest docking score (−7.84 kcal/mol) followed by acetate of norwogonin (MN8) (−7.45 kcal/mol), norwogonin (MN7) (−7.17 kcal/mol), wogonin (MN1) (−7.07 kcal/mol), methyl ether of wogonin (MN2) (−6.97 kcal/mol) and techtochrysin (MN4) (−6.8 kcal/mol). Kaempferol (MN11) and (-)-epicatechin (MN14) showed weak binding energy which was calculated as 7.63 kcal/mol and 7.86 kcal/mol, respectively.

**Figure 12:**
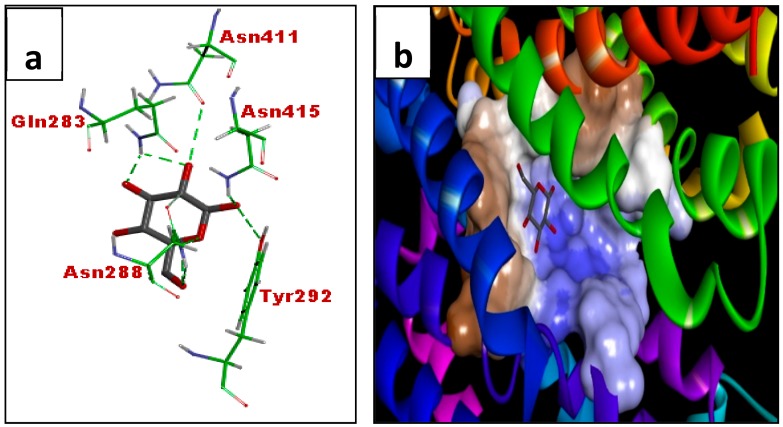
Binding interactions of glucose (**a**) with active site residues of GLUT1 (PDB ID: 5EQI). The compounds are coloured black while amino acids are green. Green and purple dashes depict hydrogen bond and hydrophobic bond, respectively. (**b**) Surface structure of glucose in active site of GLUT1. Blue and brown colours indicate polar and hydrophobic regions, respectively.

Figure 12a,b shows the active site of the GLUT1 receptor. The residues of the active sites were found to be Phe26, Trp412, Asn411, Asn415, Trp388, Gln282, Gln283, Phe379, Asn288, Phe291, and Tyr292. Among these residues, Asn411, Asn415, Gln282, Gln283, Asn288, Tyr 292 were found to interact with glucose through hydrogen bonding (Figure 12a).

**Figure 13:**
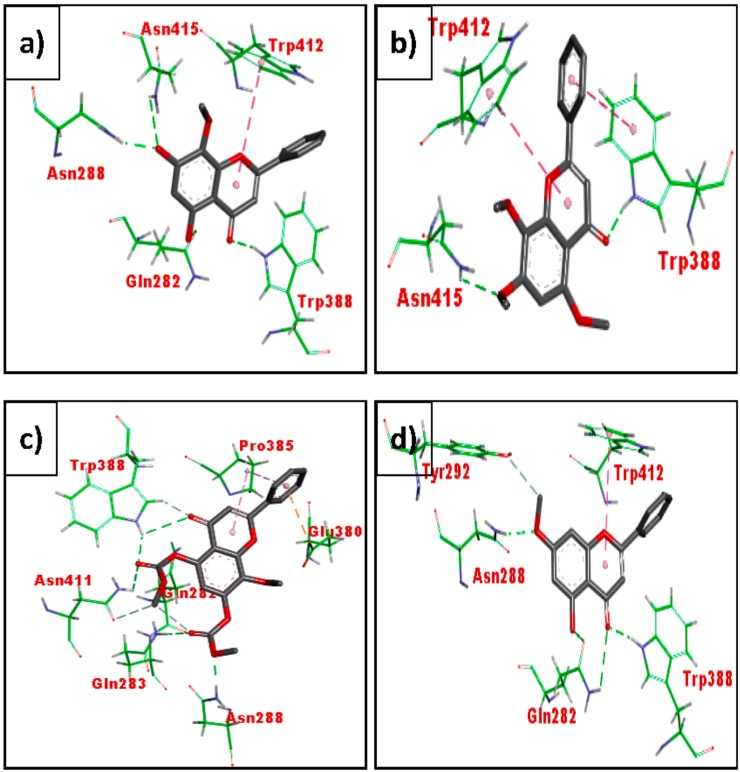
Binding interactions of wogonin (MN1) (**a**), methyl ether of wogonin (MN2) (**b**), acetate of wogonin (MN3) (**c**), and techtochrysin (MN4) (**d**) with active site residues of GLUT1 (PDB ID: 5EQI). The compounds are coloured black while amino acids are green. Green and purple dashes depict hydrogen bond and hydrophobic bond, respectively.

Figure 13 shows the binding interactions of wogonin (MN1) (a), methyl ether of wogonin (MN2) (b), acetate of wogonin (MN3) (c) and techtochrysin (MN4 (d)). All the four compounds displayed binding interactions with residues that interact with glucose in the active site, viz., Asn415, Trp412 and Trp418. Wogonin (MN1) displayed an additional interaction with Gln288 and Gln 282 while flavone-5,7,8-triacetate showed hydrogen bonding with Gln288 and Gln 282, Pro385 and Glu380.

**Figure 14:**
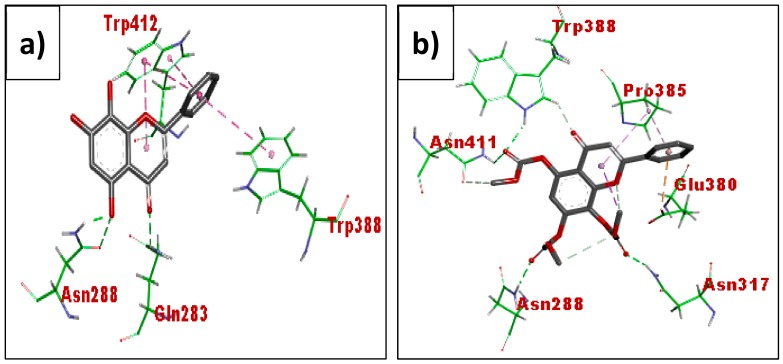
Binding interactions of norwogonin (MN7) (**a**) and acetate of norwogonin (**b**) with active site residues of Glut1 (PDB ID: 5EQI). The compounds are coloured black while amino acids are green. Green and purple dashes depict hydrogen bond and hydrophobic bond, respectively.

Acetate of norwogonin (MN8) (Figure 14) exhibited binding interactions similar to that of acetate of wogonin (MN3). It exhibited hydrogen bonding interactions with Asn411, Asn288, Trp388, Asn317, Pro385 and Glu380 while norwogonin formed hydrogen bonding with fewer amino acids which include Asn288, Gln283, trp415 and Trp388. 

**Figure 15:**
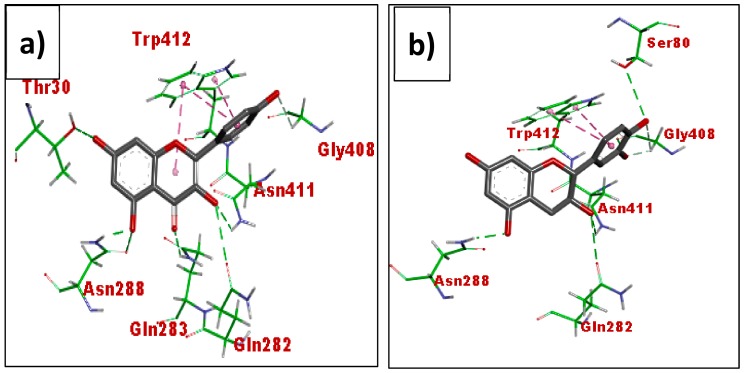
Binding interactions of kaempferol (MN11) (**a**), (-)-epicatechin (MN14) (**b**) with active site residues of Glut1 (PDB ID: 5EQI). The compounds are coloured black while amino acids are green. Green and purple dashes depict hydrogen bond and hydrophobic bond, respectively.

Figure 15 shows the binding pattern of kaempferol (MN11) (a) and (-)-epicatechin (MN14) (b), respectively. Kaempferol (MN11) displayed hydrogen bonding interaction with Asn288, Gln282, Gln283, Asn411 and Gly408 while (-)-epicatechin (MN14) exhibited hydrogen bonding with Asn288, Gln282, Asn411, ASn288, Gly408 and Ser30. Both compounds displayed hydrophobic interactions with Trp412.

## 4. Discussion

### 4.1. In Vitro Antidiabetic Evaluation on RIN-5F Pancreatic Cells and 3T3-L1 Adipocytes

Glucose is the most potent stimulator of insulin secretion and can achieve acute and long-term regulation of insulin [30]. Nevertheless, some nutrients are also able to activate insulin release or intensify glucose-stimulated insulin secretions such as hormones [31,32], proteins [33,34], and pharmacological agents [35]. In the present study, flavonoids were subjected to a RIN-5F pancreatic cell to determine the effect of plant-based compounds on insulin secretion stimulation. From the cell viability of the RIN-5F pancreatic cell, the lowest concentration of flavonoids (0.39 μg/mL) was applied due to their high cytotoxicity effect against this cell lines. The results showed that the acetate of wogonin (MN3) from the flavones group revealed the highest insulin secretion. The presence of acetate group at C-7 and methoxy group at C-8 on a flavone was found to improve the insulin secretagogue effect. Subsequently, after the acetate of wogonin, the hypolaetin (MN10) showed the highest insulin release indicative of the presence of catechol system at B ring which may be responsible as one of the factors to enhance the insulin secretagogue effect similar to glibenclamide. Jayaprakasam et al. [36] reported that stimulation of insulin secretion from rodent pancreatic β-cell is influenced by the number of hydroxyl groups at B-ring of flavonoids. Next, methyl ether of wogonin (MN2), kaempferol (MN11), (+)-catechin (MN13), acetate of norwogonin (MN8), (-)-epicatechin (MN14), quercetin (MN12), 8-hydroxy-7-methoxyflavone (MN5), chrysin (MN6), norwogonin (MN7) and isoscutellarein (MN9) exhibited moderate insulin secretagogue effect. Meanwhile, wogonin (MN1), and techtochrysin were shown to hamper insulin secretion ability resulting from the manifestation of the cytotoxicity effect against the cell lines.

Adipocytes are involved in the development of insulin resistance, resulting from the dysfunction of insulin signalling pathway [37]. Thus, improving adipocyte function and the complementation or replacement of poorly functioning adipocytes could be beneficial in T2DM [38]. Adipogenesis can be initiated by the activation of insulin signalling as well as the glucose uptake pathway, and consequently has been used for screening the natural compounds with antidiabetic activity. Results from cell viability of preadipocyte study demonstrated 25.0 μg/well as the safe dose of flavonoids (especially flavones group). Concentrations higher than 25.0 μg/well were found cytotoxic in nature which was also supported by the results demonstrated by Hasan et al. [22]. Results of lipid droplet quantification (adipogenesis) showed that at 25.0 μg/well, acetate of norwogonin (MN8) and acetate of wogonin (MN3) displayed the highest value of lipid droplet stained by oil red O that revealed about the importance of acetate group at C-5, C-7 and C-8 in these two compounds. Moreover, methoxy group at C-8 was also found to improve adipogenesis. Consequently, norwogonin (MN7), wogonin (MN1), and methyl ether of wogonin (MN2) highlighted the importance of methoxy group at C-7 and C-8 positions. Techtochrysin (MN4), 8-hydroxy-7-methoxyflavone (MN5), and chrysin (MN6) also showed good lipid droplet formations as compared to the non-treated group, (*p* < 0.05). Groups mentioned above exhibited increased in lipid droplet formation in a dose-dependent manner. On the other hand, it can be observed that isoscutellarein (MN9), hypolaetin (MN10), kaempferol (MN11), quercetin (MN12), (+)-catechin (MN13) and (-)-epicatechin (MN14) showed average lipid droplet formation, however not in a dose-dependent manner, as compared to other flavonoids groups. 

According to Shang et al. [39], glucose uptake in adipocyte is the consequence of stimulation of insulin receptor (i.e., PPARs, GLUTs) by insulin. Research study has shown that the direct uptake of 2-[*N*-(7-nitrobenz-2-oxa-1,3-diazol-4-yo)amino]-2-deoxyglucose (2-NDBG) is transported using the same glucose transporter (GLUT) as d-glucose [40] (Marin-Juez, 2015). The result showed that at 12.5 and 25 μg/mL, wogonin (MN1), methyl ether of wogonin (MN2), acetate of wogonin (MN3), techtochrysin (MN4), 8-hydroxy-7-methoxyflavone (MN5) and norwogonin (MN7) showed statistically significant glucose uptake measurement as compared to non-treated group (*p* < 0.05) that indicated the importance of methoxy group at position C-8, acetate group at position C-7 and the configuration of C-5, C-7 and C-8 positions. It is observable that these groups showed the glucose [2-NDBG] uptake in a dose-dependent manner, whereas, isoscutellarein (MN9), hypolaetin (MN10), kaempferol (MN11), quercetin (MN12), (+)-catechin (MN13), and (-)-epicatechin (MN14) did not follow the same trend of glucose uptake. This consistent trend has further validated the lipid droplet formation (adipogenesis) result. Johnstan et al. [41] reported that (-)-epicatechin demonstrated a marked reduction in glucose absorption via competitive inhibition of sodium dependent glucose transporter-1 (GLUT-1). Previously, Nomura et al. [42] reported that kaempferol and quercetin inhibited insulin signal transduction, and inhibited the translocation of GLUT4 and uptake of glucose in MC3T3-G2/PA6 adipose cells. 

For adipogenesis and 2-NDBG uptake activity, flavonols with methoxy groups showed stronger effects particularly those with a methoxy group at the C-3 position. Matsuda et al. [16] and Saito et al. [43] agreed that presence of the methoxy group in flavonoids enhance differentiation and lipolysis in 3T3-L1 adipocytes. With regard to SAR of flavonoids, it was found that flavonoids with acetate groups showed stronger effects. The existence of methoxy group also enhanced the activity particularly those with a methoxy group at the C-7 and C-8-positions. It was also found that flavonoids that possess a high number of hydroxy groups showed less activity of adipogenesis [16].

### 4.2. In Vitro Molecular Antidiabetic Evaluation

According to Dunmore and Brown [44], insulin is released from a pancreatic islet in response to increased blood glucose uptake into the adipocyte via insulin binding and signalling and further led to increased storage of *triacylglycerol*. Due to this reason, insulin resistance in adipose tissue also contributes to β-cells failure and eventually leads to T2DM. Molecules released from adipocyte (adipokines) are influenced by the flow of uptake and release from the cells. Hence, the present study investigated the adipokines measurement upon the treatment of flavonoids to find the association between glucose uptake and adipokines secretion (viz., leptin, adiponectin, TNF-α, and RBP-IV). For leptin measurement, at 12.5 μg/mL, the result of all groups of tested flavonoids showed statistically reduced leptin concentration (pg/mL) as compared to the non-treated group (*p* < 0.05). However, (-)-epicatechin (MN14) showed the lowest reduction similar to positive control, rosiglitazone (10 μM) that indicated that ketonic functional group at position C-4 in a flavonoid molecule is not essential in order to reduce the leptin concentration. This result was further followed by techtochrysin (MN4), isoscutellarein (MN9), quercetin (M12), (+)-catechin (MN13), wogonin (MN1), 8-hydroxy-7-methoxyflavone (MN5), norwogonin (MN7), chrysin (MN6), hypolaetin (MN10), kaempferol (MN11), acetate of wogonin (MN3), acetate of norwogonin (MN8), and methyl ether of wogonin (MN2), respectively, which indicated that the presence of acetate and methoxy groups slightly helped to reduce the leptin concentration.

Adiponectin is an adipocyte-derived hormone that reverses insulin resistance associated with both lipoatrophy and obesity [43]. For adiponectin measurement, at 12 μg/mL, except for acetate of norwogonin (MN8) and kaempferol (MN11), results of the tested flavonoids showed statistically increased adiponectin concentration (ng/mL) as compared to the non-treated group (*p* < 0.05) which suggested that acetate group is responsible for the inhibition of adiponectin secretion. Hypolaetin (MN10) showed the highest adiponection concentration, followed by isoscutellarein (MN9), norwogonin (MN7), (-)-epicatechin (MN14) that highlighted the role of hydroxyl groups especially in the B ring (catechol) of flavonoids. Methyl ether of wogonin (MN2), wogonin (MN1), (+)-catechin (MN13), techtochrysin (MN4), quercetin (MN12), 8-hydroxy-7-methoxyflavone (MN5), acetate of wogonin (MN3) and chrysin (MN6) showed moderate secretion of adiponectin.

Tumor necrosis factor α (TNF-α) is a well-known marker associated with induction of obesity related to insulin resistance and dyslipidemia [44]. According to Moller [45], neutralization of TNF- α is able to improve insulin sensitivity. Results showed that at 12 μg/mL, except for acetate of norwogonin (MN8), results of the tested flavonoids displayed statistically decreased TNF-α concentration (ng/mL) as compared to the non-treated group (*p* < 0.05) which revealed that the acetate group did not help the cells to suppress, whereas the hydroxyl group was able to contain TNF-α production. Quercetin (MN12) showed the lowest concentration of TNF-α, followed by (-)-epicatechin (MN14), norwogonin (MN7), kaempferol (MN11), (+)-catechin (MN13) which revealed that the total number of hydroxyl groups and configuration are essential in suppressing TNF-α in the molecules of flavonoids. Wogonin (MN1), techtochrysin (MN4), chrysin (MN6), methyl ether of wogonin (MN2) and acetate of wogonin (MN3) showed moderate TNF-α suppression which clearly indicated that the methoxy group does not possess strong impact in order to facilitate the cell to go against this marker. 

The liver and adipocytes secrete retinol-binding protein-4 (RBP-4) which affects systemic insulin sensitivity and glucose homeostasis [46]. Studies have found that serum RBP-4 level is elevated in insulin-resistant states in mice and humans [46,47]. The present study showed that at 12 μg/mL of flavonoids treatment exhibited statistically reduced in RBP-IV concentration (ng/mL) as compared to non-treated group (*p* < 0.05) except for techtochrysin (MN4). (-)-epicatechin (MN14) showed the lowest RBP-4 concentration, followed by kaempferol (MN11), hypolaetin (MN10), norwogonin (MN7), 8-hydroxy-7-methoxyflavone (MN5), methyl ether of wogonin (MN2), wogonin (MN1), isoscutellarein (MN9), chrysin (MN6), acetate of wogonin (MN3), acetate of norwogonin (MN8), quercetin (MN12), and the least concentration was displayed by (+)-catechin (MN13), respectively. These results further highlighted the essential features of the total number and configuration of hydroxyl groups in the flavonoids skeleton. Meanwhile, methoxy and acetate groups in the flavonoids molecules were also found to increase the effect of flavonoids against this adipokine.

Decrease in the translocation of GLUT-4 protein of the plasma membrane has been found to be the main cause of the insulin resistance [48]. Moreover, C/EBP-α is one of the major transcriptional factors leading to adipocyte differentiation, and regulates glucose metabolism and insulin sensitivity [49]. Although the relationship between GLUT 4 and C/EBP-α is unclear, there is a cross-regulation between both proteins during the adipogenesis process. The density index of GLUT 4 and C/EBP-α proteins was projected from the image of proteins bands on the PVDF membrane. β-actin was taken as the internal standard to normalize the proteins’ band. Among all groups, acetate of wogonin (MN3), methyl ether of wogonin (MN2) and wogonin (MN1) significantly upregulated the GLUT 4 protein (*p* < 0.05) which emphasized the importance of the methoxy group at C-8 and the acetate group at C-7 attached to the flavones that helped to trigger the GLUT4 expression. Techtochrysin (MN4) and 8-hydroxy-7-methoxyflavone (MN5) showed no significant difference with the non-treated group, while the rest of the flavonoids showed insignificant upregulated expression as compared to the non-treated group (*p* > 0.05). For the expression of C/EBP-α, 8-hydroxy-7-methoxyflavone (MN5) showed the highest expression, followed by norwogonin (MN7), acetate of norwogonin (MN8), methyl ether of wogonin (MN2), techtochrysin (MN4), wogonin (MN1), and chrysin (MN6) (*p* < 0.05). The rest of the flavonoids showed downregulation of C/EBP-α expression (*p* > 0.05). The loss of synergism between GLUT 4 and C/EBPα was observed and it can be suggested that GLUT4 and C/EBP-α have a weak interaction between each other.

### 4.3. In Silico Molecular Docking Using GLUT 1

Glucose transporter 1 (GLUT1) belongs to a family of homologous sugar transporters or cotransporters found in both prokaryotes and eukaryotes. It is a uniporter that transports glucose from the extra cellular membrane into cells [50]. GLUT1 is the ubiquitous glucose transporter in the human body and is absolutely essential for cell viability [51]. Crystallized structure of GLUT-4 is yet to be discovered. Hence, owing to the same fact, GLUT 1, an isoform of GLUT 4, was chosen for the molecular docking as a representative of the glucose transporter receptor to understand the mechanism of action of the tested flavonoids through in silico studies [52,53]. Human GLUT 1 and GLUT 4 share approximately 68.7% of amino acids identity as computed using the Biopolymer module of Tripos [54]. Flavonoids showing good glucose uptake and Western blotting effects were chosen for in silico molecular docking study. Among the eight flavonoids docking scores of GLUT 1 receptor indicated by free binding energies, acetate of wogonin (MN3) showed the highest docking score (−7.84 kcal/mol) followed by acetate of norwogonin (MN8) (−7.45 kcal/mol), norwogonin (MN7) (−7.17 kcal/mol), wogonin (MN1) (−7.07 kcal/mol), methyl ether of wogonin (MN2) (−6.97 kcal/mol) and techtochrysin (MN4) (−6.8 kcal/mol). Both kaempferol (MN11) and (-)-epicatechin (MN14) have already been reported to exert the inhibitory effect against GLUT4 [55] and therefore served as the negative control against the GLUT1 receptor in this study. The results showed weak binding energy of kaempferol and (-)-epicatechin, which were at 7.63 kcal/mol and 7.86 kcal/mol, respectively. 

Methyl ether of wogonin (MN2), acetate of wogonin (MN3) and techtochrysin (MN4) displayed binding interactions with the residues that interact with glucose in the active site, viz., Asn415, Trp412 and Trp418. Wogonin (MN1) displayed additional interaction with Gln288 and Gln 282 while flavone-5,7,8-triacetate showed hydrogen bonding with Gln288 and Gln 282, Pro385 and Glu380. The interactions of these flavonoid derivatives with Trp412 and Trp388 which was not observed in the case of glucose might have accounted for the agonist effect of these molecules towards the GLUT1 protein. Moreover, acetate of norwogonin (MN8) exhibited binding interactions similar to that of acetate of wogonin (MN3). Hydrogen bonding interactions with Asn411, Asn288, Trp388, Asn317, Pro385 and Glu380 were exhibited by acetate of norwogonin (MN8), whereas, norwogonin (MN7) formed hydrogen bonding interactions with fewer amino acids which include Asn288, Gln283, trp415 and Trp388. For kaempferol (MN11) ligand, hydrogen bonding interactions with Asn288, Gln282, Gln283, Asn411 and Gly408 were exhibited, however, for (-)-epicatechin (MN14), hydrogen bonding interactions with Asn288, Gln282, Asn411, Asn288, Gly408 and Ser30 were observed. Both compounds displayed hydrophobic interactions with Trp412. Both compounds also showed fewer similarities of bonding interaction with the co-crystallized ligand as compared with the bonding interactions of the other which explained the weak free-binding energy against the receptor. The flavonoids bind to the inner polar region of the active site. Unlike the inhibitors of GLUT1 such as cytochalasin B, which are bulky molecules that occupy the larger portion of the GLUT1 active site to exert their inhibitory effect, flavonoids are smaller molecules. This perhaps could indubitably explain why these flavonoids activated the glucose transport protein instead of exerting an inhibitory effect.

**Figure 16:**
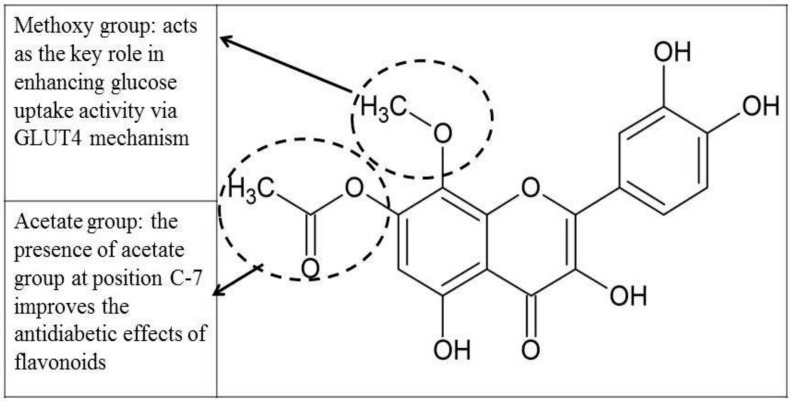
Methyl and acetate groups act as the key role in the antidiabetic activity for the glucose uptake mechanism.

From SAR perspectives, results of cell culture, molecular and in silico assays have shown that the chemical *criterion* is a fundamental for the bioactivity of these polyphenolic compounds as shown in Figure 16. The attachment of a methoxy group at position C-8 and acetate group at C-7 are the remarkable determinants for adipogenesis and glucose uptake activities via the glucose transporter (GLUT4) mechanism rather than the flavan backbone alone. Moreover, configuration of the functional group at positions C-5, C-7 and C-8 has carried significant role in antidiabetic activities. SAR of wogonin and its derivatives by [56] have earlier reported similar findings, where the methoxy group (C-8) as well as free phenol groups at positions C-5 and C-7 play very important roles for its bioactivity (anti-inflammatory activity). Meanwhile, Nicolle et al. [14] have reported that methoxy group at C-5 leads to a better antidiabetic activity of flavonoids via sodium glucose cotransporter-2 inhibitor. The present study agreed to the fact that positions C-5, C-7, and C-8 are the essential positions, and therefore these positions need to be conjugated/configured with appropriate functional groups i.e., methoxy and acetate groups to enhance the antidiabetic effect via glucose uptake activity. 

## 5. Conclusions

Flavonoids are one of the most important groups of bioactive compounds among secondary metabolites. We have reported antidiabetic activities of some selected flavonoids from in vitro and in silico perspectives. The present study concludes that tested flavonoids have shown in vitro antidiabetic activities through improved insulin secretion, upregulatory activity of glucose uptake, regulating adipokines secretion, and activation of glucose transporter (GLUT4). The results of this study have further enabled us to understand the key pharmacophores of flavonoids via a SAR study and should be encouraged for further studies, which could ultimately lead to the development of nutritional products and semi-synthetic analogs that retain substantial antidiabetic capacities with minimal adverse effects.

## Figures and Tables

**Table 1:** Docking score of selected flavonoids against GLUT 1.

Code	Ligand	Free Binding Energy (kcal/mol)
Control	Cytochalasin B (co-crystallize ligand)	−10.75
MN1	Wogonin	−7.07
MN2	Methyl ether of wogonin	−6.97
MN3	Acetate of wogonin	−7.84
MN4	Techtochrysin	−6.8
MN7	Norwogonin	−7.17
MN8	Acetate of norwogonin	−7.45
MN11	Kaempferol	7.63
MN14	(-)-Epicatechin	7.86
